# Comparison of Tracheal Intubation Using King Vision (Non-channeled Blade) and Tuoren Video Laryngoscopes in Patients With Cervical Spine Immobilization by Manual In-Line Stabilization: A Randomized Clinical Trial

**DOI:** 10.7759/cureus.43471

**Published:** 2023-08-14

**Authors:** Killo Ramesh, Gnanasekaran Srinivasan, Prasanna U Bidkar

**Affiliations:** 1 Anesthesiology and Critical Care, Jawaharlal Institute of Postgraduate Medical Education and Research, Puducherry, IND

**Keywords:** intubation difficulty score, intubation time, glottic visualization, mils, video laryngoscope

## Abstract

Background: Glottic visualization on cervical immobilization with manual in-line stabilization (MILS) might be challenging in individuals with cervical spine injuries. We compared non-channeled King Vision video laryngoscope (VL) (Ambu GmbH, Bad Nauheim, Germany) with Tuoren video laryngoscope (Henan Tuoren Medical Device, Zhengzhou, China) for endotracheal intubation in patients with cervical spine immobilization.

Methods: A total of 124 patients undergoing elective surgery under general anesthesia were included in this study. After induction of general anesthesia, patients were randomized into two groups (62 each): group K (non-channeled blade of King Vision video laryngoscope) and group T (Tuoren video laryngoscope). Cervical spine immobilization was achieved with manual in-line stabilization. The success of the first pass intubation, the time required to intubate, glottic visualization, and intubation difficulty score (IDS) were recorded.

Results: The first-attempt success rate of intubation was 95.2% (59 out of 62 patients) in group K and 90.3% (56 out of 62 patients) in group T, which were comparable. The mean glottic visualization time was significantly less with group T (12.74 ± 6.32 seconds) compared to group K (17.92 ± 4.24 seconds). Intubation time was significantly faster with group K (18.79 ± 5.857 seconds) compared to group T (27.21 ± 8.514 seconds). Both video laryngoscopes provided good grades of glottic visualization.

Conclusions: We conclude that the performance of the Tuoren video laryngoscope is similar to the King Vision video laryngoscope in terms of first-attempt intubation success rate and glottic visualization score in patients with cervical spine immobilization by manual in-line stabilization. Although glottic visualization time was shorter with Tuoren VL, we could achieve faster intubation with King Vision VL.

## Introduction

Endotracheal intubation using a direct laryngoscope requires flexion of the upper cervical spine and extension of the atlantoaxial joint, which will align the oral-pharyngeal-laryngeal axes, causing the line of vision to fall on the laryngeal inlet [1]. However, any movement of the cervical spine during laryngoscopy can worsen spinal cord injury in patients with cervical spine injuries [2]. The cervical spine can be immobilized during airway intervention with the use of manual in-line stabilization (MILS), in which the patient's occipital and mastoid processes are stabilized by an assistant [3]. MILS of the cervical spine can lead to poor glottic view, thereby rendering the airway difficult [4]. Flexible bronchoscope-guided tracheal intubation was considered the gold standard for patients with cervical spine injuries. However, video laryngoscopes (VLs) have been increasingly used in the initial airway management of these patients [5]. VL provides better visualization of the glottis and reduces cervical spine movement in cervical spine injury patients [6,7]. Compared with direct laryngoscopes, video laryngoscopes reduce the risk of intubation failure and increase first-attempt success rates [8].

King Vision VL (Ambu GmbH, Bad Nauheim, Germany) has a reusable video display and a disposable blade that can be channeled or unchanneled. King Vision VL causes much lesser movements of the C0-C1 and C3-C4 segments and has significantly improved the glottic view in cervical spine immobilization [9]. Tuoren video laryngoscope (Henan Tuoren Medical Device, Zhengzhou, China) has a detachable 3-inch light-emitting diode (LED) monitor that can be rotated to 180° and a high-intensity LED light source. Although the benefits of the various alternative devices are convincing, these devices differ with respect to first-attempt success rate, glottic visualization, and intubation time [10]. More randomized trials using MILS are warranted to determine the better airway device in cervical spine-immobilized patients. As the use of Tuoren VL has not been explored in cervical spine immobilized patients, we decided to study this device by comparing it with King Vision VL. Our primary objective was to compare the successful first-attempt intubation rate. The secondary objective was to compare the intubation time, hemodynamic response, percentage of glottis opening (POGO) score, and intubation difficulty score (IDS). We hypothesized that Tuoren VL would be associated with a better first-attempt intubation success rate due to their relatively lesser angulation.

## Materials and methods

**Figure 1:**
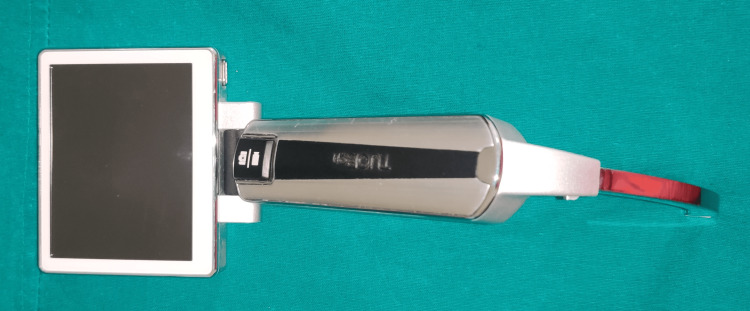
Tuoren video laryngoscope

**Figure 2:**
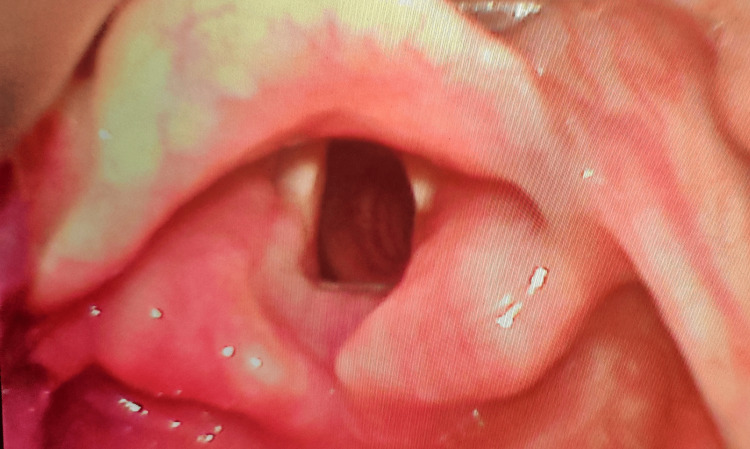
Glottic view with Tuoren video laryngoscope

We obtained approval from the Jawaharlal Institute of Postgraduate Medical Education and Research (JIPMER) Institutional Ethics Committee for institutional studies (approval number: JIP/IEC/2019/410, approval date: 30/01/2020) and registered the trial with the Clinical Trial Registry of India on 19/06/20 (CTRI/2020/06/025993). This prospective randomized trial was conducted from August 2020 to March 2021 at JIPMER, Puducherry, India. The procedures followed in our study were in accordance with the guidelines laid down in the World Medical Association (WMA) Declaration of Helsinki (ethical principles for medical research involving human subjects) [11]. We obtained informed written consent from all the participants of the study and for using the data for research and educational purposes. We enrolled 124 adult patients aged 18-70 years, American Society of Anesthesiologists (ASA) physical status classification I and II, scheduled for any elective surgery requiring general anesthesia with tracheal intubation. Patients with obesity (body mass index (BMI) of more than or equal to 30 kg/m^2^), limited neck extension, restricted mouth opening, disrupted airway anatomy, craniofacial anomaly, upper airway disease, cervical spine pathology or injury, and presence of risk factors for gastric aspiration were excluded from the study. A total of 124 patients were randomized into two groups (62 each) using a computer-generated randomization code hidden in opaque envelopes, which were serially numbered. In group K, a non-channeled blade of King Vision VL was used, and in group T, Tuoren VL was used for endotracheal intubation (Figures 1, 2).

All patients were assessed for pre-anesthetic evaluation, including a routine airway assessment, and then were randomized into one of the two groups on the day before surgery. All patients were kept nil per oral for eight hours and received anti-aspiration prophylaxis as premedication. Inside the operating room, after securing the IV line, standard monitors were attached. Preoxygenation with 100% O2 using a tight sealing mask for three minutes was done. Anesthesia was induced with fentanyl 2 mg/kg, propofol 2 mg/kg, and vecuronium 0.1 mg/kg to achieve muscle relaxation. Patients were administered isoflurane (2%) in a 50% oxygen and air mixture using a circle absorber apparatus. Video laryngoscopes and blades were checked for every case.

Just before laryngoscopy, an assistant anesthesiologist provided MILS by standing at the side of the anesthesiologist performing the intubation and grasping the mastoid process of the patient on both sides with both hands, thereby limiting any movement of the head and neck. A head ring was placed below the head for all the patients to achieve a neutral position. Group K patients were intubated using King Vision VL, and group T patients with Tuoren VL. All patients were intubated by a professionally trained anesthesiologist who conducted a minimum of 20 intubations with each device. We used a conventional endotracheal tube (ETT) with an internal diameter (ID) of 8 mm for males and a size of 7 mm for females. The ETT was pre-formed to the shape of the Tuoren VL or King Vision VL blade with the help of stylet. We used modified Cormack-Lehane (CL) grade and percentage of glottis opening (POGO) scores to evaluate the laryngeal view [12,13].

The POGO score describes how much of the glottic opening is visible during laryngoscopy. A POGO score of 100% means the entire glottic opening from the anterior commissure of the vocal cords to the interarytenoid notch is visible. A POGO score of 0% indicates no visualization of laryngeal structures.

Heart rate and mean arterial pressure (MAP) were measured at the following timelines: pre-induction, post-induction, immediately after insertion of the laryngoscope blade, and at one, three, and five minutes after successful intubation. An intubation attempt was defined as any effort to put a laryngoscope blade into the larynx, regardless of whether the endotracheal tube was successfully placed in the trachea or not. The inability to intubate within 120 seconds or two attempts was characterized as failure to intubate. In case of failure of intubation, it was decided to terminate MILS, and the anesthesiologist intubated with the usual head and neck position with a laryngoscope of their choice. The period between the insertion of the laryngoscope blade through the incisors to the optimal viewing of the glottis was termed glottic visualization time. The period from glottis visualization to the emergence of three consecutive square waveforms of the end-tidal CO2 (EtCO2) tracing on the monitor was considered intubation time. The total time taken for intubation was calculated from the moment the laryngoscope blade was inserted through the incisors until three consecutive square wave patterns appeared in the EtCO2 trace. An individual observer, who was utilized for all the study subjects, recorded the time for glottic visualization and intubation with the help of the stopwatch in the monitor (Aisys CS2, GE Healthcare, Chicago, IL, USA). The modified intubation difficulty score (IDS) (Table 1) was used to measure the difficulty of intubation with seven parameters [14,15]. Each of the seven parameters is given a score of 0 or 1, and the final value is the sum of the individual scores. An absolute IDS value of 0 is considered easy intubation, 0 to less than 5 denotes slight difficulty, and a value of more than 5 indicates moderate to major difficult intubation. The use of alternative approaches, such as the use of gum elastic bougie and optimum external laryngeal manipulation (OELM), was noted. Airway mucosal injuries, esophageal intubation, episodes of desaturation (SpO2 less than 92%), bronchospasm, and dental injury were all documented as consequences.

**Table 1:** Intubation difficulty score CL: Cormack-Lehane

Score	Parameter	Rules for calculating IDS
N1	Number of intubation attempts	Add 1 point for each supplementary attempt
N2	Number of operators	Add 1 point for each additional operator
N3	Alternative technique used	Add 1 point for each alternative technique
N4	Glottic exposure (CL grade)	Apply CL grade for first attempt. For successful intubation, N4=0
N5	Lifting force applied	Normal = 0, increased = 1
N6	External pressure applied	No = 0, yes = 2
N7	Vocal cord position at intubation	Abducted = 1, adducted = 1

The sample size was calculated by using the formula for two proportions based on the first-attempt success rate between King Vision and Tuoren video laryngoscope, and the maximum sample size was considered. Singleton et al., in a network meta-analysis, observed that the pooled probability of first-pass intubation success rate with the King Vision VL in cervical spine immobilization was 72% [16]. We expected our first-attempt intubation success rate to be 20% higher with the Tuoren video laryngoscope than with the King Vision video laryngoscope, at a 95% confidence level with a power of 80%. The sample size was calculated to be 62 patients in each group for the present study. We considered a p-value of less than 0.05 to be statistically significant.

We used Statistical Package for the Social Sciences (SPSS) version 26 (IBM Corp., Armonk, NY, USA) to conduct statistical tests. The results of continuous measures were provided as mean ± standard deviation (SD), whereas the results of categorical measurements were given as a number (percent). The significance of research parameters in two groups of patients was determined using an independent t-test. The chi-square test was used to examine the significance of study parameters on a categorical scale comparing two groups.

## Results

**Figure 3:**
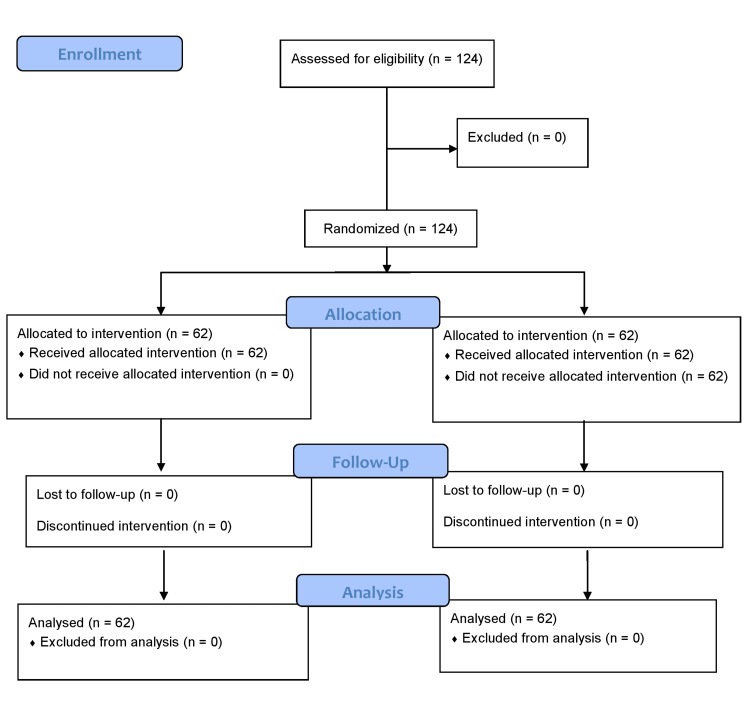
CONSORT flow diagram showing patient progress through the study CONSORT: Consolidated Standards of Reporting Trials

A total of 124 patients were randomly allocated into two groups (62 in each group), and data of all 124 patients were analyzed since none of the patients were excluded, as shown in the CONSORT flow diagram in Figure 3.

Demographic parameters were comparable except for body mass index (BMI), which was higher in group K, as shown in Table 2. Good glottic visualization was achieved in all patients (CL grades 1 and 2). No significant difference was observed in the mean POGO scores between both groups. We experienced slight difficulty in intubation (IDS 0-5) in nine patients and moderate difficulty (IDS > 5) in only one patient in group T compared to 10 patients in group K with slight difficulty and only one patient with moderate difficulty, which was not statistically significant.

**Table 2:** Demographic variables, glottic view by CL classification and POGO score, and IDS in the two groups *Statistically significant BMI: body mass index, CL: Cormack-Lehane, POGO: percentage of glottic opening, IDS: intubation difficulty score, SD: standard deviation

Variables	Group T (n = 62)	Group K (n = 62)	p-value
Age (years) (mean ± SD)	45.40 ± 12.78	41.98 ± 12.70	0.138
Weight (kg) (mean ± SD)	60.76 ± 11	61.16 ± 11.62	0.843
BMI (kg/m^2^) (mean ± SD)	24.926 ± 2.42	25.612 ± 1.48	0.047^*^
CL grade 1 (number (%))	61 (98.4%)	55 (88.7%)	0.062
CL grade 2 (number (%))	1 (1.6%)	7 (11.3%)	
POGO score (mean ± SD)	98.4 ± 1.6	88.7 ± 11.3	0.062
IDS = 0 (number (%))	52 (83.9%)	51 (82.3%)	0.969
IDS = 0-5 (number (%))	9 (14.5%)	10 (16.1%)	

Intubation was successful on the first attempt in 56 out of 62 patients in group T and 59 out of 62 patients in group K, which were comparable, as shown in Table 3. Six patients in group T and three patients in group K were successfully intubated on the second attempt. Although we achieved a significantly faster glottic visualization (p = 0.001) with Tuoren video laryngoscope (12.74 ± 6.3 seconds) when compared with King Vision VL (17.92 ± 4.2 seconds), the mean intubation time (p = 0.001) was significantly faster with King Vision VL. The total time required for intubation was significantly shorter (p = 0.037) with the King Vision VL (36.55 ± 6.5 seconds) when compared with the Tuoren VL (39.27 ± 7.7 seconds), as shown in Table 3.

**Table 3:** Success rate of first-attempt intubation and intubation times in the two groups *Statistically significant

Variables	Group T (n = 62)	Group K (n = 62)	p-value
Success rate of first-attempt intubation (number (%))	56 (90.3%)	59 (95.2%)	0.491
Glottis visualization time (seconds)	12.74 ± 6.32	17.92 ± 4.24	0.001*
Intubation time (seconds)	27.21 ± 8.51	18.79 ± 5.86	0.001*
Total time required for intubation (seconds)	39.27 ± 7.76	36.55 ± 6.58	0.037*

**Figure 4:**
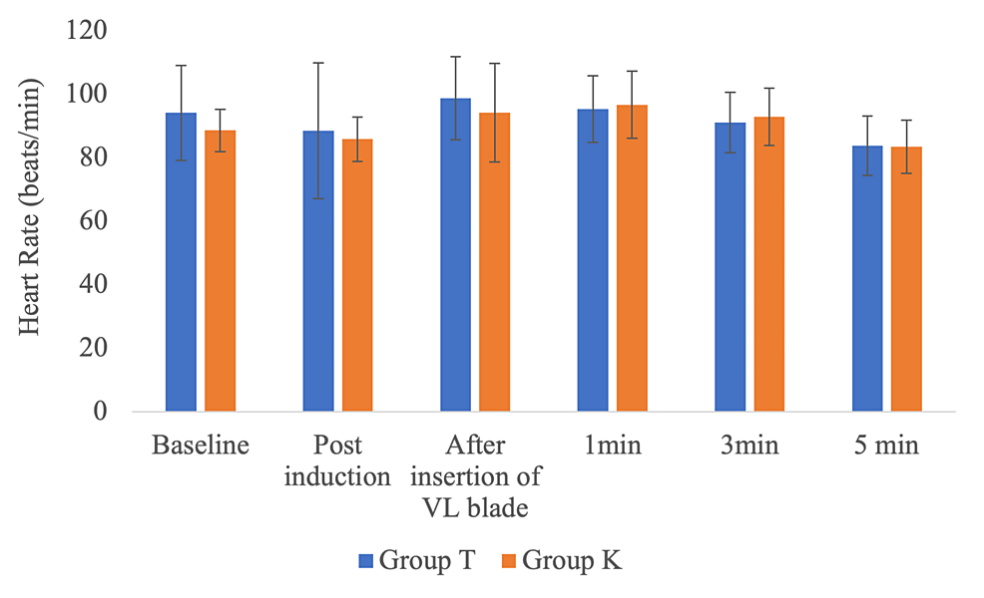
Comparison of heart rate between the two groups at different timelines Group T: Tuoren video laryngoscope group, group K: King Vision video laryngoscope group, VL: video laryngoscope

Heart rate was similar between the groups at all timelines, as shown in Figure 4.

**Figure 5:**
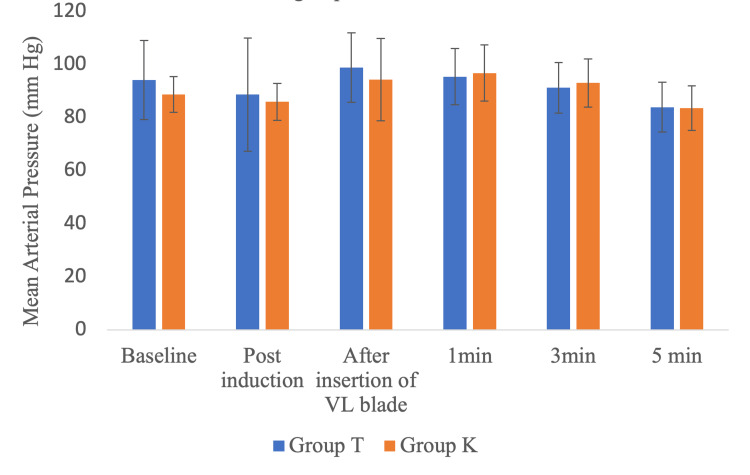
Comparison of mean arterial pressure between the two groups at different timelines Group T: Tuoren video laryngoscope group, group K: King Vision video laryngoscope group, VL: video laryngoscope

Although the pre-induction MAP values are significantly lesser in group K, MAP values at other timelines were comparable between the groups, as shown in Figure 5. None of our patients developed any airway-related complications, such as desaturation, laryngospasm, bronchospasm, and airway trauma.

## Discussion

Airway management of patients with cervical spine injuries poses a considerable challenge because of the risk of neurological damage associated with neck movements during laryngoscopy and intubation. Manual in-line stabilization of the cervical spine can negatively impact the glottic visualization and time required for successful intubation [17]. The VL blades' distinctive curvature can help provide a better view of the glottis when there is a restriction of cervical spine movement with manual in-line stabilization or the application of a rigid cervical collar [18,19]. However, this may not translate into higher successful intubation rates or faster intubation times [10,20,21].

We compared the intubation success rate and intubation parameters with Tuoren VL, a newer device, and King Vision VL when the cervical spine is immobilized. Both these devices consist of single-use disposable blades, and Tuoren VL blades are low-priced and more economical than King Vision VL blades. In our study, we achieved 100% successful intubation in all patients in both groups. The first-attempt success rate was 90.3% and 95.2% in Tuoren and King Vision video laryngoscopes, respectively, which was comparable. Glottic visualization time was significantly faster with Tuoren VL as compared to King Vision VL in our study. The prolonged time to achieve optimal visualization of the glottis with King Vision VL can be explained by its relatively lengthier handle and acute blade angle, leading to an increase in the time taken to insert it into the oral cavity when compared to the Tuoren VL blade. The curvature of the Tuoren VL blade is like that of the conventional Macintosh blade. The handle of the Tuoren video laryngoscope has a foldable video monitor, which makes the handle shorter compared to King Vision VL, in which the monitor is non-foldable, making the handle lengthier.

Our findings are very identical to those of Shravanalakshmi et al., who reported first-time success rates of 100% (45/45), 93.3% (42/45), and 95.6% (43/45) in patients who used the C blade CMAC, King Vision, and D blade CMAC video laryngoscopes [22]. All the intubations in our study were performed by experienced anesthesiologists who have completed more than 20 successful intubations with these devices, and as a result, we could achieve a good success rate of intubation.

Thiboutot et al., in a study on the effect of MILS on direct laryngoscopy, found that 46.8% of patients who received MILS had CL grade 3 laryngoscopy views [17]. In our study, we achieved lower CL grades (grades 1 and 2) in all patients with both study devices despite immobilization of the cervical spine by manual in-line stabilization. Gulati et al. compared three video laryngoscopes in patients whose cervical spine was immobilized with MILS. They observed CL grade 1 view of the larynx in 95% of patients with King Vision VL, similar to our study [23]. The CL grades observed in our study were identical to the study by Shravanalakshmi et al. in patients with cervical spine injuries, in which they showed good glottis visibility (CL grades 1 and 2) in all patients with C blade CMAC, King Vision, and D blade CMAC video laryngoscopes [22]. In addition to CL grading, we also used POGO scores to categorize the laryngeal view. POGO score is a simpler quantitative measure of the visualization of glottic opening with better inter-physician reliability than CL grading [24]. We observed a mean POGO score of 98.4% with King Vision VL, similar to previously published studies [22,25,26]. Gupta et al. reported a median POGO score of 60 (interquartile range: 50-80) with Tuoren video laryngoscope in a mannequin study in a different scenario [27]. We observed higher mean POGO scores (88.7%) with Tuoren VL, which can be explained by the better intubating conditions offered by Tuoren VL when the cervical spine is immobilized.

The total intubation time was substantially shorter with King Vision VL (36.55 ± 6.5 seconds) than with Tuoren VL (39.27 ± 7.7 seconds). Shravanalakshmi et al. showed that the conventional CMAC video laryngoscope was much quicker than the D blade CMAC video laryngoscope, while the King Vision and conventional CMAC were comparable [22]. We defined intubation time as the time from the best glottis visualization time until three consecutive square waveforms of the end-tidal CO_2_ monitor. The intubation time was significantly shorter with group K (18.79 ± 5.8 seconds) compared to group T (27.21 ± 8.5 seconds). Gupta et al. also reported faster intubation with King Vision VL when compared with Tuoren VL, although they used a channeled blade for King Vision VL, and the study was done in a mannequin in a different setting [27]. Although in our research, the Tuoren VL group had faster optimal visualization of the glottis, the total intubation time was prolonged, which could have been due to the frequent fogging of the distal lens of Tuoren VL during laryngoscopy even though we placed the blade in warm water before use.

We used the intubation difficulty score (IDS), which included seven parameters. The IDS of the two groups were comparable in our study. Our findings matched those of Shravanalakshmi et al., who found that the distribution of IDS in the D blade CMAC and King Vision VL were similar [22]. None of the patients in the groups developed any airway-related complications.

Our study has certain limitations. First, adults with normal airways who were assumed to have cervical spine injuries were included in our study. Second, we should have measured the ease of insertion of the blade and ease of intubation. Third, we did not blind the investigator to the VL type due to the study design. Fourth, the intubation difficulty scores are subjective measurements of difficult intubation. Fifth, the frequent fogging of the Tuoren VL lens could have been a confounding factor.

## Conclusions

We conclude that King Vision VL and Tuoren VL are associated with good first-attempt intubation success rates and good grades of glottic visualization in patients with cervical spine immobilization with manual in-line stabilization. Although the time to visualize the glottis was faster with Tuoren video laryngoscope, non-channeled King Vision video laryngoscope use is associated with faster intubation times in cervical spine immobilized patients. Further trials are warranted to focus on the use of these devices with hard cervical collar application.
